# Reproductive interference hampers species coexistence despite conspecific sperm precedence

**DOI:** 10.1002/ece3.7166

**Published:** 2021-02-02

**Authors:** Ryosuke Iritani, Suzuki Noriyuki

**Affiliations:** ^1^ RIKEN‐iTHEMS Wako‐shi, Saitama Japan; ^2^ Faculty of Agriculture and Marine Science Kochi University Kochi Japan

**Keywords:** community dynamics, competitive exclusion, host specialization, niche partitioning, reproductive interference, reproductive isolation, resource competition

## Abstract

Negative interspecific mating interactions, known as reproductive interference, can hamper species coexistence in a local patch and promote niche partitioning or geographical segregation of closely related species. Conspecific sperm precedence (CSP), which occurs when females that have mated with both conspecific and heterospecific males preferentially use conspecific sperm for fertilization, might contribute to species coexistence by mitigating the costs of interspecific mating and hybridization. We discussed whether two species exhibiting CSP can coexist in a local environment in the presence of reproductive interference. First, using a behaviorally explicit mathematical model, we demonstrated that two species characterized by negative mating interactions are unlikely to coexist because the costs of reproductive interference, such as loss of mating opportunity with conspecific partners, are inevitably incurred when individuals of both species are present. Second, we experimentally examined differences in mating activity and preference in two *Harmonia* ladybird species known to exhibit CSP. These behavioral differences may lead to local extinction of *H. yedoensis* because of reproductive interference by *H. axyridis*. This prediction is consistent with field observations that *H. axyridis* uses various food sources and habitats whereas *H. yedoensis* is confined to a less preferred prey item and a pine tree habitat. Finally, by a comparative approach, we observed that niche partitioning or parapatric distribution, but not sympatric coexistence in the same habitat, is maintained between species with CSP belonging to a wide range of taxa, including vertebrates and invertebrates living in aquatic or terrestrial environments. Taken together, it is possible that reproductive interference may destabilize local coexistence even in closely related species that exhibit CSP.

## Graphical abstract

We discussed whether two closely related species exhibiting conspecific sperm precedence can coexist in a local environment in the presence of reproductive interference. We used mathematical models, behavioral experiment, and comparative study. We suggested that conspecific sperm precedence does not reduce the overall cost of reproductive interference sufficiently to allow the interacting species to coexist in the same local environment.

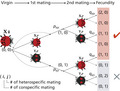

## 1 INTRODUCTION

Restrictions to local coexistence among phylogenetically related species are closely related to niche partitioning and the diversification of resource use traits, which help us understand how communities are assembled at both local and regional scales (Grant & Grant, 2011; Losos, 2011; Schluter, 2000). Therefore, understanding the mechanisms that restrict local coexistence is of fundamental importance in ecology and evolution. Reproductive interference, defined as any kind of interspecific sexual interaction that reduces the fitness of individuals (Gröning & Hochkirch, 2008), is one mechanism that can drive species exclusion at local scale and subsequent niche partitioning among species (Kyogoku & Kokko, 2020). Reproductive interference has been theoretically demonstrated to hamper species coexistence at local scale even in ecologically neutral species with similar growth rates and abilities to compete for shared resources (Crowder et al., 2011; Kawatsu, 2013; Konuma & Chiba, 2007; Kuno, 1992; Kyogoku & Sota, 2017; Nishida et al., 2015). Moreover, empirical studies have also reported that reproductive interference contributes to niche partitioning between congeneric species with overlapping mating signals, including in frogs (Ficetola & Bernardi, 2005), birds (Vallin et al., 2012), mites (Takafuji et al., 1997), and insects (butterflies, Friberg et al., 2013; grasshoppers, Hochkirch et al., 2007; ladybirds, Noriyuki et al., 2012). Therefore, reproductive interference can be a determinant of local and regional species diversity in a wide range of animal taxa in nature, though its significance in community ecology has been underestimated for decades (Gröning & Hochkirch, 2008; Kyogoku, 2015).

A number of mechanisms, however, are reported to mitigate the negative impacts of reproductive interference on the coexistence of species occupying the same niche, including plastic responses in reproductive traits (Otte et al., 2016), continued dispersal to new sets of ephemeral resource patches (Ruokolainen & Hanski, 2016), and reinforcement of reproductive isolation (Bargielowski et al., 2013). One possible mitigating mechanism is conspecific sperm precedence (CSP), where females that have mated with both conspecific and heterospecific males preferentially use conspecific sperm for fertilization (Howard, 1999). Such females might experience fewer costs associated with interspecific mating and hybridization (i.e., waste of gametes), because most or all of their offspring will be pure conspecifics (Marshall et al., 2002; Nakano, 1985; Tsuchida et al., 2019; Veen et al., 2001). In addition, in various animals, mating order has been shown to have no influence on whether a female is able to preferentially use conspecific sperm (Howard et al., 1998; Marshall et al., 2002), suggesting that complete CSP can largely eliminate the negative impact of interspecific mating provided that females have mated with at least one conspecific male before the onset of oviposition or birthing (Marshall et al., 2002). CSP has been reported in a variety of animal taxa, including crickets (Howard et al., 1998), fruit flies (Price, 1997), beetles (Fricke & Arnqvist, 2004; Rugman‐Jones & Eady, 2007), fishes (Yeates et al., 2013), mice (Dean & Nachman, 2009), and even in external fertilizers such as sea urchins (Geyer & Palumbi, 2005), mussels (Klibansky & McCartney, 2013), and thus potentially plays an important role in species coexistence. Although CSP has attracted much attention as a driver of speciation through post‐mating pre‐zygotic reproductive isolation (Howard, 1999; Howard et al., 1998), it is still unclear whether CSP can sufficiently decrease the cost of reproductive interference to promote stable coexistence of closely related species in the same local environment.

On the other hand, CSP may not fully function as a barrier against reproductive interference. Under imperfect species discrimination, individual females may incur a variety of costs as a result of interactions with heterospecific males during the reproductive process, such as reduced longevity and oviposition rates (Kawatsu & Kishi, 2017), physical damage caused by interspecific copulation (Kyogoku & Sota, 2015), and loss of opportunity to mate with conspecific partners (Noriyuki et al., 2012; Ramiro et al., 2015; Thum, 2007). CSP alone might be insufficient to compensate all of these potential costs of reproductive interference. Importantly, reproductive interference involves various behavioral mechanisms, such as mating signal jamming which might not be mitigated by CSP. In addition, adaptive behaviors of females and males can prevent multiple matings by females and consequently make the CSP mechanism useless; that is, females who mated with heterospecific male first may reject additional matings even with conspecific males, leaving ovipositing females without conspecific sperm. In fact, studies on sexual conflict have shown that females are likely to avoid multiple matings when the benefit is low (Arnqvist & Rowe, 2005; Eberhard, 1996). Moreover, to prevent sperm competition, males often try to prevent females from mating multiple times, for example, by mate guarding after copulation (Alcock, 1994), by placing a physical plug in female reproductive organs (Masumoto, 1993; Matsumoto & Suzuki, 1992; Polak et al., 2001), or by insertion of a chemical that inhibits remating receptivity (Gillott, 2003; Himuro & Fujisaki, 2008; Scott, 1986). Therefore, to evaluate the ecological role of CSP in species coexistence, various behavioral and physiological mechanisms affecting the reproductive process must be taken into account.

**FIGURE 1:**
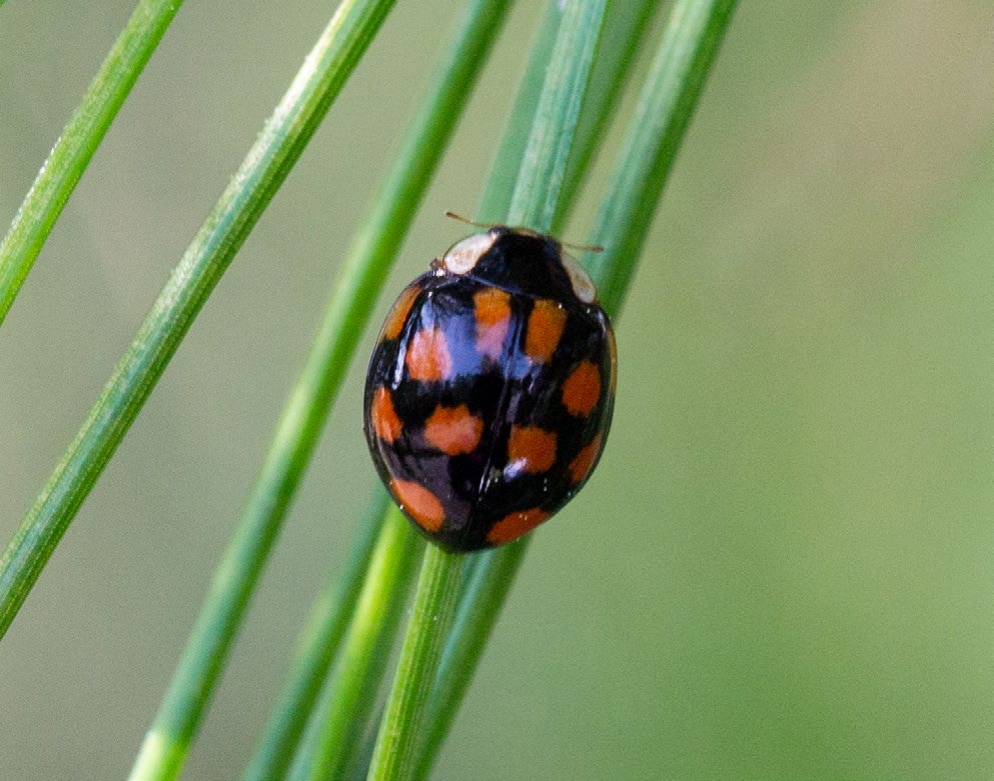
An adult of *Harmonia yedoensis*. Both *H. yedoensis* and *H. axyridis* show genetic polymorphism on body color pattern

In this study, we discussed whether CSP can mitigate the effect of reproductive interference in two species so that they are able to coexist in a local environment. We adopted a tripartite approach. First, we developed a behaviorally explicit mathematical model to analyze behavioral and demographic factors affecting local species coexistence, with a focus on the multiple copulation, mating preference toward conspecific or heterospecific partners, and the initial population densities of the two species. Second, we conducted mating experiments with two predatory ladybird species, *Harmonia axyridis* and *Harmonia yedoensis* (Figure 1), especially focusing on the effect of mating experience on the additional matings behavior. CSP has been detected in both these species (Noriyuki et al., 2012), and they occupy different habitats in nature (i.e., realized niches); *H. axyridis* is a generalist that feeds on various species of aphids, whereas *H. yedoensis* specializes on the giant pine aphid, which is a highly elusive prey item and nutritionally poor for larval development (Noriyuki & Osawa, 2012; Noriyuki et al., 2011). In addition, the reproductive success of *H. yedoensis* females is strongly decreased in the presence of *H. axyridis* males, suggesting that the effect of reproductive interference might be highly asymmetric and *H. yedoensis* might utilize the less preferred food and habitat to avoid reproductive interference from *H. axyridis* (Noriyuki et al., 2012). Third, we investigated the general consequences of CSP on species coexistence in nature by compiling published data on pairs of species in which CSP has been detected and found that such species pairs generally show niche separation (habitat and food source) or geographically separate distributions. We suggested from our results that CSP does not reduce the overall cost of reproductive interference sufficiently to allow the interacting species to coexist in the same local environment.

## 2 MATERIALS AND METHODS

### 2.1 Mathematical model

We modeled a community of two species (X and Y), with density $N_{X}\left(t\right)$ and $N_{Y}\left(t\right)$, respectively, at time t, inhabiting a single patch. The two species interact through resource competition as well as through reproductive interference, but they are ecologically neutral in terms of fecundity per capita (denoted *r* ≥ 1), density‐dependent mortality (denoted v), and interspecific resource competition (denoted *b*). We assumed a sex ratio of 1:1 (though we found that the ratio does not affect the results; see Kyogoku & Sota, 2017), and, for the sake of simplicity, at most two instances of copulation per female (see Appendix S1: Supporting Information A for the general case when copulation can occur more than twice). Finally, we assumed that females are not always capable of correctly assessing the species identity of their mating partner; as a result, interspecific mating can occur even after intraspecific mating (as is the case in *H. yedoensis* and *H. axyridis*, Noriyuki et al., 2012).

**FIGURE 2:**
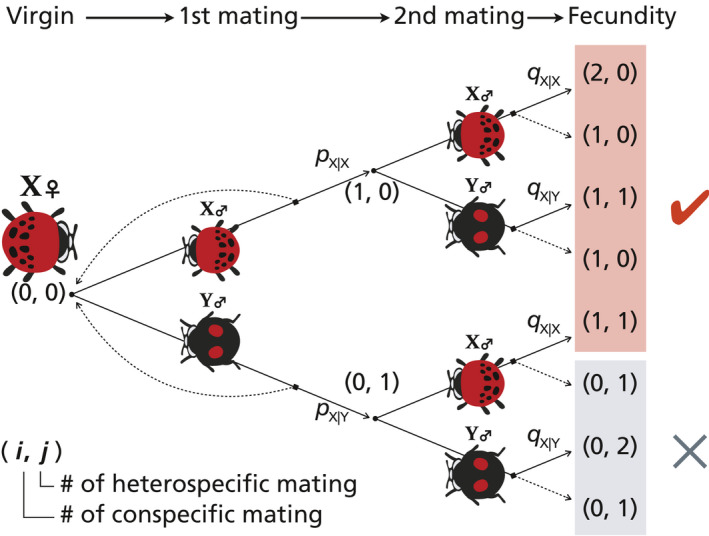
Schematic mating decision‐making tree for a female of species X according to our mathematical model. Here, (i,j) means a female with *i* intraspecific matings and *j* interspecific matings (1≤i+j≤2). A virgin female has state (0,0), and she accepts a given X‐male or Y‐male with a probability $p_{X|X}$ and $p_{X|Y}$, respectively. Subsequently, the non‐virgin female with state (0, 1) or (1, 0) accepts an X‐male or Y‐male with probability $q_{X|X}$ or $q_{X|Y}$, respectively. The corresponding mating decision‐making tree for a Y‐female can be obtained by exchanging X and Y. The female states after the second mating that include at least one intraspecific mating (*i* ≥ 1) are shaded red; in this case, the female can produce offspring of her own species through CSP. The states of females that failed to copulate with a conspecific male before producing offspring (*i* = 0) are shaded gray

Species X and Y differ in the rate at which females accept males as mates, depending on their mating status and counterpart species (Figure 2; we call the diagram as mating decision‐making tree). Specifically, an unmated X‐female (i.e., a female of species X) accepts a mating attempt by an X‐male with probability $p_{X|X}$ and a Y‐male with probability $p_{X|Y}$, and a once‐mated female accepts a mating attempt by an X‐male with probability $q_{X|X}$ and with a Y‐male with probability $q_{X|Y}$. We assumed *p* and *q* values as different parameters because females are likely to reject multiple matings in some species (see Introduction). Similarly, the probabilities of a Y‐female accepting a mating attempt by a male in the corresponding situations are $p_{Y|Y},p_{Y|X},q_{Y|Y}$, and $q_{Y|X}$.

We restricted our model to two‐shots mating in the main analysis. As we explained in the Introduction, the number of mating in females is limited by several reasons in many animal species. In *H. axyridis* females, for example, multiple mating was not common in the field (Osawa, 1994). Therefore, we assumed that (a) a female mates with conspecific or heterospecific male after any number of rejection behavior in the first phase of the mating decision‐making tree and that (b) some females reject multiple mating before oviposition in the second phase (Figure 2). However, we also considered the general case that mating occurs more than twice (see below).

We denote the expected fecundity by a single X‐female or a single Y‐female by $E_{X}$ or $E_{Y}$, respectively. We let a parameter, c, tune the degree of interspecific niche overlap and reflects how frequently the two species meet in the same locality where reproductive interference can take place (0≤c≤1). Specifically, we assume that a female is subject to the choice between conspecific and heterospecific males (Figure 2) with a probability of c (or else no heterospecific mating occurs); thus, the expected reproductive output of an X‐ or Y‐female is calculated as:

(1) $$E_{X}\left(N_{X},N_{Y}\right)=\left(\left(1‐c\right)+c\cdot \frac{\left(p_{\text{X|X}}+p_{X|Y}\cdot q_{X|X}\frac{N_{Y}}{N_{X}+N_{Y}}\right)N_{X}}{N_{X}\cdot p_{X|X}+N_{Y}\cdot p_{X|Y}}\right)r,$$

$$E_{Y}\left(N_{X},N_{Y}\right)=\left(\left(1‐c\right)+c\cdot \frac{\left(p_{Y|Y}+p_{Y|X}\cdot q_{Y|Y}\frac{N_{X}}{N_{X}+N_{Y}}\right)N_{Y}}{N_{Y}\cdot p_{Y|Y}+N_{X}\cdot p_{Y|X}}\right)r,$$

(see Appendix S1: Supporting Information A for derivation). Within the parentheses on the right side of each Eqs. (1), (1‐*c*) represents reproductive success independent of density and frequency, and the second term represents the product of the degree of niche overlap (*c*) and the conditional probability that, given a non‐virgin, a single female mates with a conspecific male at least once.

We model the population dynamics under intra‐ and interspecific competition using ordinary differential equations. We separate the timescales between mating phase and community dynamics with density‐dependent mortality, by calculating the stationary probability that a female copulates with a at least one conspecific (the fraction term in Eqn 1), during which phase, no density‐dependent mortality occurs (quasi‐stationarity assumption). Under this quasi‐stationarity assumption, the dynamics are as follows:

(2) $$\frac{dN_{X}}{dt}=\left(E_{X}\left(N_{X}\left(t\right),N_{Y}\left(t\right)\right)‐N_{X}\left(t\right)‐bN_{Y}\left(t\right)\right)N_{X}\left(t\right),$$

$$\frac{dN_{Y}}{dt}=\left(E_{Y}\left(N_{X}\left(t\right),N_{Y}\left(t\right)\right)‐N_{Y}\left(t\right)‐bN_{X}\left(t\right)\right)N_{Y}\left(t\right),$$

where *b* (0≤b≤1) tunes the relative strength of interspecific resource competition. All variables and parameters are defined in Table 1. The community equilibrium is obtained by setting the RHS of Eq. (2) to zero. We then carry out a basic local stability analysis of the equilibrium of Eq. (2) to determine possible equilibrium states (see below). Specifically, we identified conditions leading to *species exclusion* (i.e., only one species persists) or *coexistence* (i.e., both species coexist).

**TABLE 1:** Parameters included in the model

Parameter/Variable	Definition	Default value (if any)
X or Y	Species label	–
$N_{X},N_{Y}$	Density of X or Y	Dynamic variable
*t*	Time (*t* ≥ 0)	–
*r*	Egg production (per capita)	25
*v*	Density dependence of resource competition (*v* > 0)	1
*b*	Strength of interspecific competition (*b* > 0)	0.3
c	Niche overlap (0 < *c* < 1); probability that a female is subject to the mating decision‐making tree	Varied
$p_{i|j}$	Probability that a virgin female of species *i*) accepts a mating attempt by a male of species *j*, where *i* and *j* can be either X or Y	See Figure 2
$q_{i|j}$	Probability that a non‐virgin female of species *i* accepts a mating attempt by a male of species *j*, where *i* and *j* can be either X or Y	See Figure 2
$f_{X}\left(=N_{X}/\left(N_{X}+N_{Y}\right)\right)$	Frequency of species X in the population	Dynamic variable
$E_{X},E_{Y}$	Expected reproductive output (per capita), calculated based upon the mating decision‐making tree (see Figure 1), for species X or Y	–

We also visualized the steady states by a numerical approach, first (i) evaluating the eigenvalues of the Jacobi matrix of equilibria and then (ii) depicting the phase portraits (using Mathematica 11.3; Wolfram Research, 2018). For the eigenvalue analyses, we first checked the number of feasible equilibria ($N_{X},N_{Y}\geq 0$) of the community dynamics (Eq. 2) and then numerically evaluated the signs of the real parts of the eigenvalues associated with the corresponding equilibria. That is, we numerically carried out a standard local stability analysis. (iii) When there are multiple stable equilibria, we numerically obtained basins of attraction by tracking the fate of each point (as an initial condition) on $\left(N_{X},N_{Y}\right)$‐plane.

We also extended the model to the general case in which the number of mating is arbitrary, typically more than twice (Appendix S1: Supporting Information A). We then found that, as the possible number of mating increases, the probability that a given female copulates with at least one conspecific male tends to approach 1, implying that CSP eliminates the negative impact of interspecific mating (Appendix S1: Supporting Information A).

### 2.2 Behavior experiment

Detailed procedure for ladybird collection and rearing was described in Appendix S1: Supporting Information B. In the mating experiment, we kept one female (*H. yedoensis* or *H. axyridis*) and one male (*H. yedoensis* or *H. axyridis*) together in a small Petri dish (5 cm in diameter) on a laboratory bench at room temperature (25°C) under constant fluorescent lighting. We never placed females with sibling males (i.e., individuals produced by the same wild‐caught mother or from the same wild‐collected clutch) to preclude any effects of inbreeding avoidance on mating behavior. We observed the occurrence of male mating attempts, female rejection behavior, and successful copulation in each experimental session (see Noriyuki et al., 2012 for the definition of these behaviors). In 2014, we visually observed mating activities during 15‐min sessions (hereafter “short experiment”). In 2015, we used video cameras (HC‐V480, Panasonic, Osaka, Japan) to record experimental sessions for at least 6 hr (up to 20 hr) and then watched the videos to analyze mating behaviors (hereafter “long experiment”). In the short experiments, each pair was allowed to mate after the 15‐min session until copulation was completed. In the long experiments, multiple copulations were allowed in the same experimental session, although the first mating occurred soon after the beginning of each experimental session in most cases. In both short and long experiments, we reused virgin and non‐virgin individuals after the experimental session for other sessions to analyze the effects of mating experience on subsequent mating behavior.

To examine the effect of mating experience in virgins and non‐virgins on the copulation rate in each species, we analyzed the proportion of experimental sessions that included at least one successful copulation by a generalized linear model with a binomial error structure using the glm function of the R software package (version 3.4.2, R Core Team, 2017). Similarly, we compared the mating rate between intra‐ and interspecific mating trials in virgin and non‐virgin females. Moreover, we analyzed mating preferences of both males and females to determine factors responsible for the copulation rate. First, we evaluated male preference by the proportion of experimental sessions that included at least one male mating attempt, whether or not it was followed by successful mating. Second, we examined the female preference by calculating the proportion of male mating attempts that elicited female rejection behavior. In all analyses, we also incorporated adult age, body length, and elytra color (black or red) of females and males as fixed effects. We analyzed the interaction effects between species identity and the main factor in each analysis (mating experience or intra‐ and interspecific mating). We analyzed data from the short and long experiments separately because of the differences in the source populations and the specific experimental conditions.

Furthermore, we applied signal detection theory to disentangle the mechanism of decision‐making in males and females who need to choose conspecific mating partner over heterospecifics (Green & Swets, 1966; Shizuka & Hudson, 2020). We computed two statistics, *d*′ and *β*, where *d*′ is signal strength (a higher value indicates that the mating signal from conspecifics is more readily detected), and *β* reflects an individual's mating strategy. *β* ≈ 1.0 indicates unbiased decision‐making; *β* ≈ 0.0 indicates a bias toward mating with either a conspecific or heterospecific individual (i.e., a liberal strategy); and *β* > 1.0 indicates a bias toward rejection of mating with either a conspecific or heterospecific individual (i.e., a conservative strategy). *d*′ and *β* in response to signals (male mating attempt and female rejection behavior) in each species were computed by using the dprime function of the neuropsychology library for the R software package (Makowski, 2017). To visualize the decision‐making performance in response to both male mating attempts and female rejection behavior, we calculated the receiver operating characteristic (ROC) curve, which compares the sensitivity (the true positive rate, plotted on the *y*‐axis) with the specificity (the false positive rate, plotted on the *x*‐axis), for the signal detection results by using the ROCR package for R (Sing et al., 2005). Essentially, the closer an ROC curve is to the upper left corner, the better the decision‐making accuracy, and the closer the curve is to the diagonal line of the panel (i.e., *y* = *x*), the more likely that the result is owing to chance alone (Carter et al., 2016). Note that the shape of the ROC curve is not affected by an individual's decision criteria (*β*), although it depends on a discriminability between signal and noise (*d*′). In addition, we used the DeLong method in the pROC package for R (Robin et al., 2011) to statistically compare the area under the ROC curve (AUC) between species in each experiment year.

### 2.3 Comparative study

We performed a literature survey, using the ISI Web of Science (https://webofknowledge.com/) on 30 November 2017 and the key phrase “conspecific sperm precedence,” to identify congeneric pairs of animal species in which CSP had been detected in at least one of the pair. In addition, we screened the reference lists of two review papers for CSP (Howard, 1999; Marshall et al., 2002) to locate additional pairs. We classified the geographic distributions and niches of each pair into one of four categories: (a) sympatry, geographical distribution of the two species largely overlaps with little if any niche separation in the sympatric area; (b) niche partitioning, geographical distributions of the two species overlap with niche partitioning at local scale (e.g., separation by food, habitat, or seasonality) especially at the reproductive stage; (c) parapatry, geographical distributions of the two species do not overlap but are adjacent with a narrow contact (hybridization) zone; or (d) allopatry, geographical distributions of the two species do not overlap and are not adjacent. We excluded species with cosmopolitan, human‐mediated distributions (e.g., *Drosophila simulans*, *Tribolium* flour beetles, and *Callosobruchus* bean weevils) from the analysis because their habitats and distributions in the natural environment are unclear. In addition, although it is possible that unidentified cryptic species were included in the study systems, we adopted species taxonomy based on the searched publications on CPS. In total, we analyzed 24 species pairs of marine invertebrates, terrestrial insects, and vertebrates.

## 3 RESULTS

### 3.1 Mathematical model

#### 3.1.1 Equilibria

We found dynamic population equilibria, designated by an asterisk (*), on (i) the $N_{X}$‐axis (i.e., $N_{X}^{*}>0,N_{Y}^{*}=0$), (ii) the $N_{Y}^{*}$‐axis (i.e., $N_{X}^{*}=0,N_{X}^{*}>0$), or (iii) in the interior (i.e., $N_{X}^{*}>0,N_{Y}^{*}>0$). The boundary equilibria (as a result of species exclusion) are given by

(3) $$B_{X}=\left(r,0\right),B_{Y}=\left(0,r\right)$$

whereas the interior equilibrium did not have analytical formula.

### 3.2 Stability analyses

The stability conditions for the equilibria (species exclusion or coexistence) were determined from the eigenvalues of the Jacobi matrix around the focal equilibrium (more details are given in Appendix S1: Supporting Information C). The necessary condition for the species exclusion to be stable is given by:

(4) b+c>1

**FIGURE 3:**
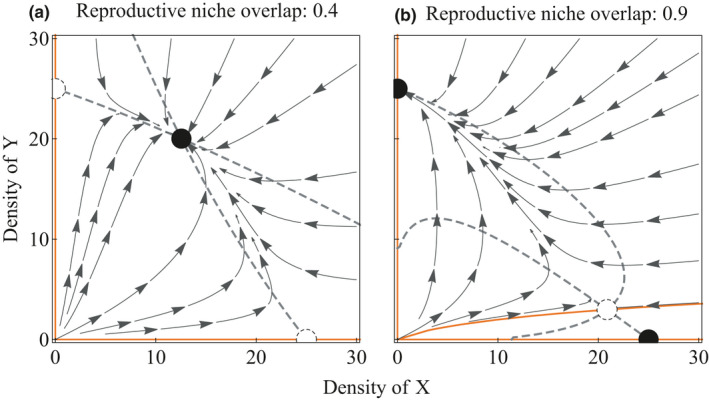
Phase portraits of the approximated, time‐continuous dynamics (i.e., ODE; Eq (3), for varying niche overlap (tuned by c). (a) Coexistence is possible when niche overlap is low (c=0.4). (b) Species exclusion occurs when niche is largely overlapped between the two species (c=0.9). Dashed curves: isoclines; arrows: phase portraits; solid curves: boundaries of basins of attraction (separatrix); open circles: unstable equilibria; and closed circles: stable equilibria. The procedure used to produce the figures is described in Supporting Information D. Probability parameter values: $p_{\mathrm{XX}}=0.4$, $q_{\mathrm{XX}}=0.4,p_{\mathrm{XY}}=0.8,p_{\mathrm{YY}}=0.8,p_{\mathrm{YX}}=0.4,q_{\mathrm{YY}}=0.8$; other parameters, default values (see Table 1)

From this species, exclusion becomes more likely as c increases (Figure 3); specifically, the reproductive interference can lead in the community to species exclusion that was otherwise of coexistence. See Appendix S1: Supporting Information D for the numerical procedures for visualization. Moreover, see Kishi and Nakazawa (2013) for the interactive effects of resource competition and reproductive interference on species exclusion in detail.

We note here that, if the two species are highly symmetric in terms of *p* and *q* values, then more outcomes become possible; in particular, species exclusion and coexistence states can be stable simultaneously (“bi‐stable”), in agreement with Kishi and Nakazawa (2013) and Kyogoku and Sota (2017). Our particular intention here, however, was to explore the effects of asymmetry in mating behavior (*p* and *q* values) on the community dynamics in our experimental system. For more details about the consequences of symmetric *p* and *q* values, see Appendix S1: Supporting Information E. Also, it is possible to incorporate differences in the number of mating attempts in a given time period (i.e., mating activity) such that the encounter rate with an X‐ or Y‐male can be biased toward either species relative to their frequency in the community; however, changes in the encounter rate did not change the results dramatically, although species exclusion became more likely (see Appendix S1: Supporting Information E for more information).

### 3.3 Experiment

**FIGURE 4:**
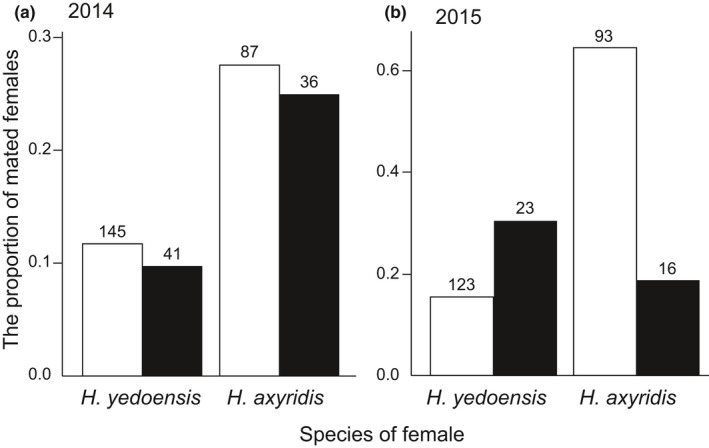
The proportion of mated females with conspecific (white) and heterospecific (black) males in the (a) short (2014) and (b) long (2015) experiments. The number of individuals in each category is shown above each bar

Mating experience did not have a significant effect on the rate of copulation in either the short (*z* = 1.601, *p* = .109) or the long experiment (*z* = 0.943, *p* = .346); therefore, virgin and non‐virgin females were pooled in the following analyses. In the long experiment, *H. axyridis* was more likely to mate with conspecifics, whereas no such assortative mating pattern was observed in *H. yedoensis*; that is, there was a significant interaction effect between female species and species identity of the mating partner (*z* = –3.692, *p* < .01), although there was not such significant interaction effect in the short experiment (*z* = 0.460, *p* = .645; Figure 4). In both the short and long experiments, *H. axyridis* males more frequently attempted to mate with conspecific females, whereas *H. yedoensis* males did not show such a strong preference toward conspecific females (interaction effects between male and female species, short: *z* = –3.480, *p* < .01; long: *z* = –1.742, *p* = .08). *Harmonia axyridis* females were more likely than *H. yedoensis* females to refuse mating attempts by conspecific males, especially in the short experiment (interaction effects between male and female species, short: *z* = –2.054, *p* < .05; long: *z* = –0.019, *p* = .985); however, both coercive mating and copulation failure occurred in both species following female rejection behavior.

**FIGURE 5:**
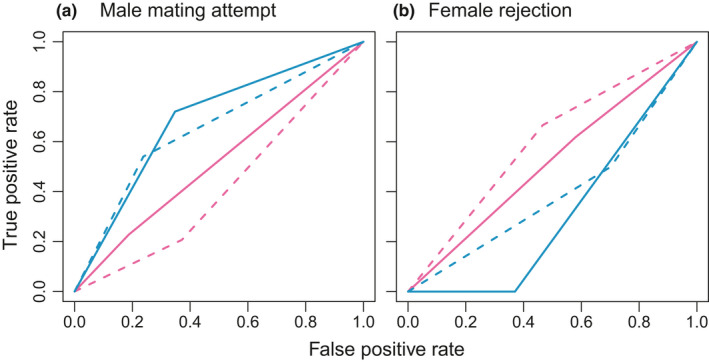
Receiver operating characteristic curves for (a) mating attempts by males and (b) rejection behavior in females. In each panel, red and blue lines indicate *H. yedoensis* and *H. axyridis*, respectively, and dashed and solid lines indicate the short and long experiments, respectively

In the signal detection analysis results, *d*′ of male mating attempts was higher in *H. axyridis* than in *H. yedoensis* in both short and long experiments (Table 2). Further, the AUC for male mating attempts was significantly higher in *H. axyridis* than in *H. yedoensis* in both short and long experiments (Figure 5, Table 3). These results mean that *H. axyridis* males are likely to distinguish conspecific from heterospecific females for their mating partner, whereas *H. yedoensis* males promiscuously mate with both conspecific and heterospecific females. By contrast, the AUC result for female rejection behavior was also not significantly different between species, probably in part because of the small sample size (Table 4).

**TABLE 2:** Results for the signal detection theory indices

Behavior	Year	Species	*d*′	*β*
Male mating attempt	2014	*H. yedoensis*	–0.480	0.760
		*H. axyridis*	0.794	1.274
	2015	*H. yedoensis*	0.099	1.081
		*H. axyridis*	0.960	0.921
Female rejection	2014	*H. yedoensis*	0.490	1.095
		*H. axyridis*	–0.487	0.871
	2015	*H. yedoensis*	0.098	1.026
		*H. axyridis*	–0.441	1.271

**TABLE 3:** Area under curve (AUC) and its statistical comparison between species. Significant results are shown in bold

Behavior	Year	AUC		Statistics		
		*H. yedoensis*	*H. axyridis*	*D*	*df*	*p*
Male mating attempt	2014	0.429	0.635	–3.738	298.130	**<.001**
	2015	0.512	0.630	–2.247	220.750	**.026**
Female rejection	2014	0.571	0.421	1.648	87.723	.103
	2015	0.519	0.485	0.481	52.513	.632

**TABLE 4:** The number of behavioral responses to correct signal (conspecific individuals) and false signal (heterospecific individuals) in mating attempt of males and rejection behavior of females. Hit and Correct Rejection indicates right response to correct and false signals, respectively. Miss and False Alarm indicates wrong response to correct and false signals, respectively

Behavior	Year	Species	Correct signal		False signal	
			# Hit	# Miss	# False Alarm	# Correct rejection
Male mating attempt	2014	*H. yedoensis*	30	115	13	22
		*H. axyridis*	47	40	10	32
	2015	*H. yedoensis*	28	95	3	13
		*H. axyridis*	67	26	8	15
Female rejection	2014	*H. yedoensis*	16	14	3	6
		*H. axyridis*	14	33	7	7
	2015	*H. yedoensis*	13	18	8	13
		*H. axyridis*	97	57	3	0

### 3.4 Comparative study

We found spatial separation at both local (niche partitioning) and regional scales (parapatry or allopatry) among species pairs exhibiting CSP, including in marine abalones, freshwater fishes, terrestrial insects, birds, and mice (Table 5). We observed parapatry mainly in Orthoptera (crickets and grasshoppers). We detected sympatry without apparent niche partitioning in 6 of 24 species pairs, especially in aquatic invertebrates such as mussels, starfishes, and sea urchins.

**TABLE 5:** Summary of comparative study results

Group	Common name	Species pair	Category	Description	References
Marine invertebrate	Abalone	*Haliotis corrugata* and *H. rufescens*	Sympatry	Niche overlap in terms of water depth and habitat	1–3
		*Haliotis cracherodii* and *H. rufescens*	Niche partitioning	Intertidal zone versus kelp forest habitat	3, 4
		*Haliotis fulgens* and *H. rufescens*	Niche partitioning	Shallow versus deep water habitats	2, 3, 5
	Blue mussel	*Mytilus trossulus* and *M. edulis*	Sympatry	Hybrid zone is not narrow	6, 7
	Starfish	*Asterias forbesi* and *A. rubens*	Sympatry	Similar habitats, food resources, and spawning time	8, 9
	Coral	*Montastraea annularis* and *M. franksi*	Niche partitioning	Separation in (slightly overlapped) spawning time	10
	Sea urchin	*Echinometra mathaei* and *E. oblonga*	Sympatry	Slight ecological differences	11
		*Echinometra oblonga* and *E*. sp. C	Sympatry	Slight difference in habitat but similar spawning time	12
Terrestrial invertebrate	Cricket	*Allonemobius fasciatus* and *A. socius*	Parapatry		13, 14
		*Gryllus firmus* and *G. pennsylvanicus*	Parapatry		15, 16
		*Gryllus bimaculatus* and *G. campestris*	Parapatry		17, 18
	Grasshopper	*Chorthippus p. parallelus* and *C. p. erythropus*	Parapatry		19, 20
		*Podisma pedestris* races	Parapatry		21, 22
	Ladybird	*Epilachna pustulosa* and *E. vigintioctomaculata*	Niche partitioning	Host plant separation	23, 24
		*Harmonia yedoensis* and *H. axyridis*	Niche partitioning	Difference in prey item and habitat	25
	Fruit fly	*Drosophila yakuba* and *D. santomea*	Parapatry	Lowland versus highland distributions	26, 27
	Stalk‐eyed fly	*Teleopsis dalmanni* diverged populations	Allopatry		28, 29
	Damselfly	*Ischnura graellsii* and *I. elegans*	Niche partitioning	The two species are rarely found in the same localities	30–33
Vertebrate	Darter fish	*Etheostoma barrenense* and *E. zonale*	Sympatry	Not closely related within the genus	34
		*Etheostoma hopkinsi* and *E. luteovinctum*	Allopatry		35
	Salmonid	*Salmo* salar and *S. trutta*	Niche partitioning	Spatial and temporal segregation in spawning activities	36–38
	Sunfish	*Lepomis macrochirus* and *L. gibbosus*	Niche partitioning	Differences in nesting and breeding habits	39, 40
	Bird	*Ficedula hypoleuca* and *F. albicollis*	Niche partitioning	Separation in breeding habitat	41–43
	Mouse	*Mus domesticus* and *M. musculus*	Parapatry		44, 45

Note

1: Vacquier et al. (1990); 2: Cox (1962); 3: Lindberg (1992); 4: Vacquier and Lee (1993); 5: Kresge et al. (2000); 6: Klibansky and McCartney (2014); 7: Gaitán‐Espitia et al. (2016); 8: Harper and Hart (2005); 9: Menge (1979); 10: Fogarty et al. (2012); 11: Metz et al. (1994); 12: Geyer and Palumbi (2005); 13: Howard et al. (1998); 14: Howard and Waring (1991); 15: Larson et al. (2012); 16: Harrison and Arnold (1982); 17: Tyler et al. (2013); 18: Veen et al. (2013); 19: Butlin (1998); 20: Butlin and Hewitt (1985); 21: Hewitt et al. (1989); 22: Hewitt (1975); 23: Nakano (1985); 24: Matsubayashi and Katakura (2009); 25: Noriyuki et al. (2012); 26: Chang (2004); 27: Lachaise et al. (2000); 28: Rose et al. (2014); 29: Christianson et al. (2005); 30: Sánchez‐Guillén, et al. (2005); 31: Sánchez‐Guillén, et al. (2011a); 32: Sánchez‐Guillén et al. (2011b); 33: Sánchez‐Guillén et al. (2013); 34: Williams and Mendelson (2014); 35: Mendelson et al. (2007); 36: Yeates et al. (2013); 37: Heggberget et al. (1988); 38: Jonsson and Jonsson (2009); 39: Immler et al. (2011); 40: Osenberg et al. (1992); 41: Veen et al. (2001); 42: Qvarnström et al. (2009); 43: Vallin et al. (2012); 44: Dean and Nachman (2009); 45: Payseur et al. (2004).

## 4 DISCUSSION

Our mathematical model highlighted the behavioral mechanisms that affect species exclusion even in the presence of CSP. The model results showed that differences in mating activity, male mating preference, and female remating acceptance determine which of two interacting species is superior with respect to reproductive interference (Appendix S1: Fig. A1, A2), whereas previous theoretical studies on reproductive interference did not fully take into account the consequences of behavioral processes on population dynamics and species' fates (Kishi & Nakazawa, 2013; Kyogoku & Sota, 2017; Yoshimura & Clark, 1994). In particular, our analysis means that CSP had little chance to function as the mechanism that secures female fertility when females sometimes refuse mating attempts by conspecific males after heterospecific mating. In addition, we found that species exclusion is more likely to occur for a wide range of initial population densities of the two species when their niche is largely overlapped (Figure 3). This finding means that closely related species are unlikely to coexist in the same environment if they have similar mating signals or if they share a niche in space and time; as a result, niche partitioning or geographical segregation of the species may occur as the previous theoretical work indicated (Nishida et al. 2015). Overall, our model supports the hypothesis that reproductive interference is likely to hamper local coexistence even in closely related species that exhibit CSP.

Our experiment using *Harmonia* ladybirds, combined with their niche partitioning in the field, will be an interesting example for the prediction of our theoretical analysis of species exclusion. The rate of copulation was not significantly different between virgin and non‐virgin females in the two *Harmonia* species. This indicates that females who mated with heterospecific male first were unlikely to reject additional matings with conspecific males, resulting CSP mechanism may able to work after multiple mating. However, the results of our GLMs and signal detection analysis indicated that *H. axyridis* males distinguish and choose conspecific females over heterospecific females, whereas mating rates with conspecifics were low in *H. yedoensis* (Figures 4 and 5). From these results, it is suggested that *H. axyridis* is likely to mate with a conspecific partner at least once before oviposition begins, whereas *H. yedoensis* females, even though they exhibit CSP, are incapable of producing viable offspring in the presence of *H. axyridis* males (Noriyuki et al. 2012). It would be interesting if *H. yedoensis* is excluded by *H. axyridis* from the local patch due to their mating interactions, as our mathematical model indicated (Figure 3). This prediction is consistent with the niche partitioning observed in the field, where *H. axyridis* feeds on preferred prey items on various types of trees and *H. yedoensis* specializes in highly elusive prey on only pine trees. The pine habitat may function as a refuge for *H. yedoensis*, where it can largely avoid reproductive interference from *H. axyridis* (Noriyuki et al. 2012).

Our comparative study found a separation of niche use or geographical distributions between some species pairs with CSP in a range of taxa (Table 5). Although we were not able to perform quantitative approach, it may be an interesting possibility that CSP alone may not allow these species pairs to coexist in the same local environment. However, sympatric coexistence without apparent niche separation was also detected, especially in free‐spawning marine invertebrates such as mussels, starfishes, and sea urchins (Table 5). There are several possible reasons that can account for the actual pattern in nature. First, niche separation might actually exist, but, perhaps because of limited field survey data, it may not have been recognized. In fact, fine‐scale differences in adult habitat and the timing of spawning have been detected in closely related marine invertebrate species (Fogarty, 2012; Lindberg, 1992). Therefore, it is possible that niche separation has actually occurred to mitigate the cost of reproductive interference in such species. Second, dispersal to new patches can allow overlapping niche use at a local scale even when two species engage in competitive interactions. Especially in marine sessile invertebrates that have high dispersal ability in the larval stage and a sedentary life style in the adult stage, source**–**sink dynamics (Mouquet & Loreau, 2003) and stochastic processes (Paine & Levin, 1981) likely promote local species coexistence. Third, in marine sessile animals, eggs may be likely to experience numerous encounters with both conspecific and heterospecific sperms in waters, which means that CSP makes it possible for females to produce viable offspring, as our general model with arbitrary number of mating demonstrated (Appendix S1: Supporting Information A). In this situation, therefore, CSP can indeed mitigate the cost of interspecific mating and thus promote species coexistence in the same niche. Clearly, it is important to incorporate life‐history characteristics and specific biology of each species when considering the community‐level consequences of behavioral decision‐making in animals.

By including plants, it would be possible to extend our model to more general scenarios of interacting species under imperfect species recognition. Reproductive interference occurs in flowering plants when the stigma receives heterospecific as well as conspecific pollen grains, for example when flowering phenology and pollinators overlap (Matsumoto et al., 2010; Nishida et al., 2014; Runquist & Stanton, 2013; Takakura, 2013). In some cases, however, conspecific pollen tubes preferentially grow and fertilize the ovules (Baldwin & Husband, 2010, reviewed in Howard, 1999). This phenomenon is called conspecific pollen precedence and is considered a mechanism of reproductive isolation that prevents hybridization, and consequently, promotes speciation in plants (Howard, 1999). Therefore, it is suggested that conspecific pollen precedence in plants, similar to CSP in animals, can mitigate the cost of reproductive interference and lead to species coexistence in the same habitat. Alternatively, as our model predicted, conspecific pollen precedence may be insufficient to allow interacting species to coexist in the same local environment. In fact, in three species of *Iris*, conspecific pollen precedence has been detected together with habitat differences (Carney et al., 1996; Emms et al., 1996), suggesting that reproductive interference destabilizes local coexistence of these species. In future, it would be interesting to examine whether our model is applicable to plant species by investigating reproductive success in species pairs exhibiting conspecific pollen precedence.

In conclusion, our study clarified the *ecological* significance of CSP by identifying conditions that lead to local species exclusion despite the presence of CSP. This finding is in contrast to those of previous studies of CSP, which have focused on its *evolutionary* significance, that is, speciation through post‐mating pre‐zygotic reproductive isolation. Moreover, many CSP studies have not quantified pre‐mating behaviors that can affect the reproductive success of females but have instead examined the functioning of CSP by focusing on post‐mating pre‐zygotic mechanisms. Importantly, however, it has been documented that the overall costs of reproductive interference, including loss of mating opportunity and decreases in the oviposition rate due to male interference, can lead to the extinction of one of the interacting species even if interspecific mating and insemination do not occur (Carrasquilla & Lounibos, 2015; Friberg et al., 2013; Kishi et al., 2009). Therefore, to understand individual reproductive success and community structure of closely related species, various behavioral and physiological mechanisms of reproductive interference, especially pre‐mating behaviors, should not be neglected.

## CONFLICT OF INTEREST

The authors declare no conflict of interest.

## AUTHOR CONTRIBUTION

**Ryosuke Iritani:** Formal analysis (lead); Visualization (equal); Writing‐original draft (equal); Writing‐review & editing (equal). **Suzuki Noriyuki:** Conceptualization (equal); Formal analysis (supporting); Investigation (lead); Methodology (lead); Visualization (equal); Writing‐original draft (equal); Writing‐review & editing (equal).

## Supporting information

Appendix S1

## Data Availability

These data and R scripts are available at https://doi.org/10.5061/dryad.vt4b8gtps (Iritani & Noriyuki, 2020).
