# Supplementary material for: Reproductive interference hampers species coexistence despite conspecific sperm precedence
Source: Ecol Evol. 2021 Feb 2;11(5):1957–69. doi: 10.1002/ece3.7166 (PMC7920778; doi:10.1002/ece3.7166)
Supplement: Supplementary file 1 — Appendix S1 [file ECE3-11-1957-s001.pdf]

A Reproductive output

Derivation of Eqs 1 and 2

We assume that a female in state-(i,j) is such that mated with a X-male i-times, and with a Y-male j-times. For instance, a state-$(0,0)$ females are unmated. Let $Q_{(i,j)}$ be the frequency of (i,j)-state X-females after the stage of mating.

Let $f_{\mathrm{X}} = 1 - f_{\mathrm{Y}} = N_{\mathrm{X}}/(N_{\mathrm{X}} + N_{\mathrm{Y}})$ be the frequency of X; the (2,0)-state is a set of females that mated with conspecifics twice, which occurs via: (i) rejecting males k-times successively (which occurs with probability ξ_k), (ii) encountering a X-male (which occurs with f_{X}), (iii) accepting him (which occurs with $p_{\mathrm{X|X}}$), (iv) again encountering a X-male (which occurs with f_{X}), and (v) accepting him (which occurs with $q_{\mathrm{X|X}}$). Bearing in mind the "loop" present in the diagram in the main text, we can show that:

$$\xi_k = \Bigg(\underbrace{f_{\mathrm{X}}}_{\text{encountering X-male}} \times \underbrace{(1 - p_{\mathrm{X|X}})}_{\text{rejecting him}} + \underbrace{f_{\mathrm{Y}}}_{\text{encountering Y-male}} \times \underbrace{(1 - p_{\mathrm{X|Y}})}_{\text{rejecting him}} \Bigg)^k = \big(1 - \overline{p_{\mathrm{X}}}\big)^k , \tag{A1}$$

where $\overline{p_{\mathrm{X}}} := f_{\mathrm{X}} p_{\mathrm{X|X}} + f_{\mathrm{Y}} p_{\mathrm{X|Y}}$ represents the average acceptance rate of an unmated X-female. At the $(k+1)$-st and $(k+2)$-nd mating trials, she accepts the mating attempts from conspecifics, which occurs with probability $f_{\mathrm{X}} p_{\mathrm{X|X}} \cdot f_{\mathrm{X}} q_{\mathrm{X|X}}$. Hence the frequency of $(2,0)$-females reads:

$$Q_{(2,0)} = \sum_{k=0}^{\infty} \xi_k f_{\mathrm{X}} p_{\mathrm{X|X}} f_{\mathrm{X}} q_{\mathrm{X|X}} = \frac{p_{\mathrm{X|X}} f_{\mathrm{X}}^2 q_{\mathrm{X|X}}}{\overline{p_{\mathrm{X}}}} = \frac{p_{\mathrm{X|X}} f_{\mathrm{X}}}{f_{\mathrm{X}} p_{\mathrm{X|X}} + f_{\mathrm{Y}} p_{\mathrm{X|Y}}} \cdot f_{\mathrm{X}} q_{\mathrm{X|X}}, \tag{A2}$$

which is interpreted as the conditional probability that a random female, given she accepted a male of either species in the first mating, has accepted a conspecific male, times the probability that in the second round of copulation, she encounters a conspecific male and accepts him.

Similarly, we get:

$$\begin{aligned} Q_{(1,1)} &= \frac{f_{\mathrm{X}} p_{\mathrm{X|X}} f_{\mathrm{Y}} q_{\mathrm{X|Y}} + f_{\mathrm{Y}} p_{\mathrm{X|Y}} f_{\mathrm{X}} q_{\mathrm{X|X}}}{f_{\mathrm{X}} p_{\mathrm{X|X}} + f_{\mathrm{Y}} p_{\mathrm{X|Y}}}, \\ Q_{(1,0)} &= \frac{f_{\mathrm{X}} p_{\mathrm{X|X}} f_{\mathrm{X}} (1 - q_{\mathrm{X|X}}) + f_{\mathrm{X}} p_{\mathrm{X|X}} f_{\mathrm{Y}} (1 - q_{\mathrm{X|Y}})}{f_{\mathrm{X}} p_{\mathrm{X|X}} + f_{\mathrm{Y}} p_{\mathrm{X|Y}}}, \\ Q_{(0,1)} + Q_{(0,2)} &= 1 - \big(Q_{(2,0)} + Q_{(1,1)} + Q_{(1,0)}\big). \end{aligned} \tag{A3}$$

Note that we are not required to explicitly evaluate the quantity on the final line, because the females in state $(0,1)$ and $(0,2)$ have not received conspecific sperms and thus have null fecundity.

From this, the expected reproductive output of a X-female per capita, E_X, is given by:

$$\begin{aligned} E_X &= (1-c)r + cr\left(Q_{(2,0)} + Q_{(1,1)} + Q_{(1,0)}\right) \\ &= (1-c)r + cr\frac{f_X}{f_X p_{X|X} + f_Y p_{X|Y}}\left(p_{X|X} + p_{X|Y} q_{X|X} f_Y\right), \end{aligned} \tag{A4}$$

where r represents the total number of offspring surviving to maturation, per capita. Switching the subscripts X and Y on each symbol supplies the corresponding quantities for a Y-female. If we substitute $f_X = 1 - f_Y = N_X/(N_X + N_Y)$, we get the expression of E_X, E_Y (as functions of (N_X, N_Y)) in the main text; we denote these by $E_X(N_X, N_Y)$, $E_Y(N_X, N_Y)$.

Altogether,

$$\begin{aligned} \frac{\mathrm{d}N_X(t)}{\mathrm{d}t} &= \Big(E_X\big(N_X(t), N_Y(t)\big) - vN_X(t) - bvN_Y(t)\Big)N_X(t) =: W_X\big(N_X(t), N_Y(t)\big), \\ \frac{\mathrm{d}N_Y(t)}{\mathrm{d}t} &= \Big(E_Y\big(N_X(t), N_Y(t)\big) - vN_Y(t) - bvN_X(t)\Big)N_Y(t) =: W_Y\big(N_X(t), N_Y(t)\big). \end{aligned} \tag{A5}$$

We can, without loss of generality, assume $v = 1$ by subsuming v with the densities (otherwise transform the densities (N_X, N_Y) to new variables $(U_X, U_Y) = (vN_X, vN_Y)$ to obtain the equivalent dynamical system).

Arbitrary numbers of mating

We have so far restricted ourselves to considering each female copulates at most twice. Here we extend the models to a case where the number of mating is arbitrary. To simplify the analyses, we assume that not all females mate at least once (as opposed to the analyses of the main text that assumes that all females do so).

In this subsection, we use the following symbols:

- M_X: density of males of species X;
- $\mu_X = 1 - \mu_Y = M_X/(M_X + M_Y)$: frequency of conspecific males for X among all males;
- F_X: total density of females of species X;
- $\varphi_X = 1 - \varphi_Y = F_X/(F_X + F_Y)$: frequency of conspecific females for X among all females;
- $F_X^{(i,j)}$: density of females of X that mated with conspecifics i times and with heterospecifics j times;
- $H_X = \sum_{j=0} F_X^{(0,j)}$: density of females of X who have mated with no conspecifics ($i = 0$) but potentially with heterospecific male ($j \geq 0$);
- $C_X = \sum_{i\geq 1, j\geq 0} F_X^{(i,j)} (= F_X - H_X)$: density of females of X who have mated with at least one conspecifics ($i \geq 1$) and potentially with heterospecific male ($j \geq 0$);
- $p_{X|X}^{(j)}$, $p_{X|Y}^{(j)}$: probability that a female species X accepts copulation from a male of species X or Y, respectively, in the j-th times of mating (for $j \geq 0$).

Demographic (community) dynamics for M_X and F_X

ODE for M_X, F_X is given by:

$$\begin{aligned} \frac{\mathrm{d}M_\mathrm{X}}{\mathrm{d}t} &= \frac{1}{2}E_\mathrm{X}\cdot F_\mathrm{X} - (N_\mathrm{X} + bN_\mathrm{Y})M_\mathrm{X}, \\ \frac{\mathrm{d}F_\mathrm{X}}{\mathrm{d}t} &= \frac{1}{2}E_\mathrm{X}\cdot F_\mathrm{X} - (N_\mathrm{X} + bN_\mathrm{Y})F_\mathrm{X}, \end{aligned} \tag{A6}$$

where $1/2$ accounts for sex ratio. From this,

$$\frac{\mathrm{d}(M_\mathrm{X} - F_\mathrm{X})}{\mathrm{d}t} = \underbrace{(N_\mathrm{X} + bN_\mathrm{Y})}_{>0}(M_\mathrm{X} - F_\mathrm{X}), \tag{A7}$$

in which quasi-stationarity gives $M_\mathrm{X} = F_\mathrm{X}$. We hereafter make use of this quasi-stationarity assumption. By summing the two lines in Eqn (A6), we get:

$$\begin{aligned} \frac{\mathrm{d}(M_\mathrm{X} + F_\mathrm{X})}{\mathrm{d}t} &= E_\mathrm{X}\cdot F_\mathrm{X} - (N_\mathrm{X} + bN_\mathrm{Y})(M_\mathrm{X} + F_\mathrm{X}) \\ &= \frac{1}{2}E_\mathrm{X}\cdot (M_\mathrm{X} + F_\mathrm{X}) - (N_\mathrm{X} + bN_\mathrm{Y})(M_\mathrm{X} + F_\mathrm{X}), \end{aligned} \tag{A8}$$

and if we subsume the factor $1/2$ to the r in E_X of Eqn (A4), we recover Eqn (A5) as $F_\mathrm{X} + M_\mathrm{X} = N_\mathrm{X}$. Therefore Eqn (A6) is consistent with the eq (1) of the main text.

Mating dynamics for $F_\mathrm{X}^{(0,j)}$

We first derive the community dynamics of females in state $(0, j)$. The following diagram depicts the dynamical structure (transitions):

$$\begin{array}{ccccc} F_\mathrm{X}^{(0,j-1)} & & & & \\ & \searrow^{\mu_\mathrm{Y} p_\mathrm{Y}^{(j-1)}} & & & \\ & & F_\mathrm{X}^{(0,j)} & \xrightarrow{\mu_\mathrm{X} p_\mathrm{X}^{(j)}} & F_\mathrm{X}^{(1,j)} \\ & & & \searrow^{\mu_\mathrm{Y} p_\mathrm{Y}^{(j)}} & \\ & & & & F_\mathrm{X}^{(0,j+1)} \end{array} \tag{A9}$$

where quantities by the arrows are transition rates per unit time per capita. From this, using a parameter δ tuning the timescale, the dynamics for $F_\mathrm{X}^{(0,j)}$ for $j \geq 1$ reads:

$$\frac{\mathrm{d}F_\mathrm{X}^{(0,j)}}{\mathrm{d}t} = \mu_\mathrm{Y} p_\mathrm{Y}^{(j-1)} F_\mathrm{X}^{(0,j-1)} - \left(\mu_\mathrm{X} p_\mathrm{X}^{(j)} + \mu_\mathrm{Y} p_\mathrm{Y}^{(j)}\right) F_\mathrm{X}^{(0,j)} - \delta(N_\mathrm{X} + bN_\mathrm{Y}) F_\mathrm{X}^{(0,j)} \tag{A10}$$

with the 'renewal' process for $j = 0$ (unmated females):

$$\frac{\mathrm{d}F_\mathrm{X}^{(0,0)}}{\mathrm{d}t} = E_\mathrm{X} \times F_\mathrm{X} - \left(\mu_\mathrm{X} p_\mathrm{X}^{(0)} + \mu_\mathrm{Y} p_\mathrm{Y}^{(0)}\right) F_\mathrm{X}^{(0,0)} - \delta(N_\mathrm{X} + bN_\mathrm{Y}) F_\mathrm{X}^{(0,0)} \tag{A11}$$

where:

$$E_\mathrm{X} := r\Big(1 - c + c \times \big(\text{Proportion females having mated with at least one conspecific}\big)\Big) \tag{A12}$$

(see Eqns (A3) and (A4)), which is equal to:

$$\begin{aligned} E_\mathrm{X} &= r\Big(1 - c \times \big(\text{Proportion females having mated with no conspecific}\big)\Big) \\ &= r\left(1 - c \times \frac{H_\mathrm{X}}{F_\mathrm{X}}\right), \end{aligned} \tag{A13}$$

which we substitute into Eqn (A11) to obtain:

$$\frac{\mathrm{d}F_\mathrm{X}^{(0,0)}}{\mathrm{d}t} = r\left(1 - c\frac{H_\mathrm{X}}{F_\mathrm{X}}\right) F_\mathrm{X} - \left(\mu_\mathrm{X} p_\mathrm{X}^{(0)} + \mu_\mathrm{Y} p_\mathrm{Y}^{(0)}\right) F_\mathrm{X}^{(0,0)} - \delta(N_\mathrm{X} + bN_\mathrm{Y}) F_\mathrm{X}^{(0,0)}. \tag{A14}$$

The first term is reproduction, the second is the sum of transitions from $(0,0)$ to $(1,0)$ and $(0,1)$, and the third is mortality (we can omit this term by separating the timescale from demographic processes; detailed below) due to competition for resource with any individuals in the community (across sexes). Thus it may be easier to derive $H_\mathrm{X} = \sum_{j=0} F_\mathrm{X}^{(0,j)}$.

To gain some ideas, let us look at the dynamics for $F_\mathrm{X}^{(0,1)}$:

$$\frac{\mathrm{d}F_\mathrm{X}^{(0,1)}}{\mathrm{d}t} = \mu_\mathrm{Y} p_\mathrm{Y}^{(0)} F_\mathrm{X}^{(0,0)} - \left(\mu_\mathrm{X} p_\mathrm{X}^{(1)} + \mu_\mathrm{Y} p_\mathrm{Y}^{(1)}\right) F_\mathrm{X}^{(0,1)} - \delta(N_\mathrm{X} + bN_\mathrm{Y}) F_\mathrm{X}^{(0,1)}, \tag{A15}$$

which, at quasi-stationarity, gives:

$$F_\mathrm{X}^{(0,1)} = \frac{\mu_\mathrm{Y} p_\mathrm{Y}^{(0)}}{\mu_\mathrm{X} p_\mathrm{X}^{(1)} + \mu_\mathrm{Y} p_\mathrm{Y}^{(1)} + \delta N_\mathrm{X} + \delta b N_\mathrm{Y}} \cdot F_\mathrm{X}^{(0,0)}, \tag{A16}$$

from which induction gives:

$$F_{\mathrm{X}}^{(0,j)} = F_{\mathrm{X}}^{(0,0)} \prod_{k=1}^{j} \left(\frac{\mu_{\mathrm{Y}} p_{\mathrm{Y}}^{(k-1)}}{\mu_{\mathrm{X}} p_{\mathrm{X}}^{(k)} + \mu_{\mathrm{Y}} p_{\mathrm{Y}}^{(k)} + \delta N_{\mathrm{X}} + \delta b N_{\mathrm{Y}}} \right), \tag{A17}$$

thus yielding:

$$\begin{aligned} H_{\mathrm{X}} &= \sum_{j=0}^{+\infty} F_{\mathrm{X}}^{(0,j)} \\ &= F_{\mathrm{X}}^{(0,0)} \sum_{j=0}^{+\infty} \prod_{k=1}^{j} \left(\frac{\mu_{\mathrm{Y}} p_{\mathrm{Y}}^{(k-1)}}{\mu_{\mathrm{X}} p_{\mathrm{X}}^{(k)} + \mu_{\mathrm{Y}} p_{\mathrm{Y}}^{(k)} + \delta N_{\mathrm{X}} + \delta b N_{\mathrm{Y}}} \right), \end{aligned} \tag{A18}$$

where $\prod_{k=1}^{0}(\cdot)$ is interpreted as 1 (power of zero). This expression is not generally valid because the infinite summation may diverge depending on the values of ps.

For analytical tractability, assuming $p_{\mathrm{X}}^{(k)} \equiv p_{\mathrm{X}}$, $p_{\mathrm{Y}}^{(k)} \equiv p_{\mathrm{Y}}$ (for any k) gives much simpler calculation. For any j (or for any $F_{\mathrm{X}}^{(0,j)}$), the probability (per unit time) of a female in that state turns into $F_{\mathrm{X}}^{(1,j)}$ is $\mu_{\mathrm{X}} p_{\mathrm{X}}$. Thus,

$$\frac{\mathrm{d}C_{\mathrm{X}}}{\mathrm{d}t} = \mu_{\mathrm{X}} p_{\mathrm{X}} H_{\mathrm{X}} - \delta(N_{\mathrm{X}} + bN_{\mathrm{Y}})C_{\mathrm{X}}, \tag{A19}$$

which at quasi-stationarity yields:

$$C_{\mathrm{X}} = \frac{\mu_{\mathrm{X}} p_{\mathrm{X}}}{\delta N_{\mathrm{X}} + \delta b N_{\mathrm{Y}}} H_{\mathrm{X}}. \tag{A20}$$

Therefore,

$$\begin{aligned} \frac{H_{\mathrm{X}}}{C_{\mathrm{X}} + H_{\mathrm{X}}} &= \frac{1}{\frac{\mu_{\mathrm{X}} p_{\mathrm{X}}}{\delta N_{\mathrm{X}} + \delta b N_{\mathrm{Y}}} + 1} \\ &= \frac{\delta N_{\mathrm{X}} + \delta b N_{\mathrm{Y}}}{\mu_{\mathrm{X}} p_{\mathrm{X}} + \delta N_{\mathrm{X}} + \delta b N_{\mathrm{Y}}}, \end{aligned} \tag{A21}$$

which, with Eqn (A14), implies:

$$\frac{\mathrm{d}F_{\mathrm{X}}^{(0,0)}}{\mathrm{d}t} = r\left(1 - c \times \underbrace{\delta \cdot \frac{N_{\mathrm{X}} + bN_{\mathrm{Y}}}{\mu_{\mathrm{X}} p_{\mathrm{X}} + \delta N_{\mathrm{X}} + \delta b N_{\mathrm{Y}}}}_{\text{mating with no consp.}}\right) F_{\mathrm{X}} - \left(\mu_{\mathrm{X}} p_{\mathrm{X}}^{(0)} + \mu_{\mathrm{Y}} p_{\mathrm{Y}}^{(0)}\right) F_{\mathrm{X}}^{(0,0)} - \delta(N_{\mathrm{X}} + bN_{\mathrm{Y}}) F_{\mathrm{X}}^{(0,0)}. \tag{A22}$$

However, the factor in the first open parenthesis is 1 minus the strength of reproductive interference (c) times the probability of mating with no conspecifics, and if we separate the timescales between community dynamics and mating dynamics by letting $\delta \to +0$, then the effect of dynamical processes on mating success tends to zero.

Intuitively, the results suggest that if each female has many chances of mating, then she certainly ends up with mating with at least one conspecific male before the onset of oviposition, but if there is a possibility that females and males may be subject to natural death during the time span of mating ($\delta \not\approx 0$), mating success may be reduced. Hence, conspecific sperm precedence can reduce the impacts of interspecific mating when mating phase is a fast process. Nevertheless, as we analyzed in the main text, conspecific sperm precedence should not fully function as a barrier against reproductive interference when the number of mating is restricted to a few times.

B Ladybird collection and rearing

We collected adults of two ladybird species from Japanese red pine (Pinus densiflora Sieb. et Zucc.) at the Kumagaya campus of Rissho University (139° 36'E, 36° 10'N) and the Hirose Wild Birds Forest (139° 35'E, 36° 14'N), Kumagaya city, central Japan, during April 2015. In the laboratory, we maintained females individually in plastic Petri dishes (9 cm in diameter by 1.5 cm high) at 25°C, and fed them each day with a surplus of frozen Ephestia kuehniella Zeller eggs (Beneficial Insectary, Ontario, Canada) to set them for reproduction. In total, 10 *H. yedoensis* and 9 *H. axyridis* females produced a sufficient number of egg clutches for our experiments. In addition, we collected 32 °H. yedoensis egg clutches and 41 *H. axyridis* egg clutches that had been oviposited on the leaves and branches of Japanese red pine trees at the Hirose Wild Birds Forest. We fed the hatched offspring from both laboratory-laid and wild-collected egg clutches with a mixture of sucrose, dried yeast, and powdered drone honeybee (following Niijima *et al.* 2000) to the adult stage in plastic cases (each 12.5 cm in diameter by 9.5 cm high) containing wood wool as a substrate on which they could walk. We recorded the date of emergence, body length (to the nearest 0.01 mm), and elytra colour (black or red) of all newly emerged adults as possible factors affecting mating preference, and used these virgin individuals for the behavioural experiments to standardize the mating experience. Because it takes approximately 1 month for most individuals of both *H. yedoensis* and *H. axyridis* to mature sexually after they emerge as adults (Okada *et al.* 1978), we reared the newly emerged adults individually in plastic Petri dishes for at least 30 days, providing them with frozen *E. kuehniella* eggs every other day, before using them in mating experiments. In addition, we excluded egg clutches from the wild-caught mothers that produced only female offspring (one *H. axyridis* female in 2015) because they were likely to be infected with male-killing bacteria (Noriyuki *et al.* 2014; Noriyuki 2016), to avoid any confounding effects of male-killing bacteria on the host mating behaviour (Majerus 2003).

C Stability analysis

The dynamical system has three boundary solutions: (i) no species (denoted $D^* = (N_X, N_Y) = (0, 0)$, in which case f_X and f_Y are undefined, so we omit this possibility by presuming that the initial condition is sufficiently distant from D^* and that r is sufficiently large); (ii) only X persists ($B^*_X = (r, 0)$), and (iii) only does Y ($B^*_Y = (0, r)$).

We here examine the stability of (ii) and (iii).

B_X^* is locally stable if the following conditions are satisfied:

$$\left.\frac{\partial W_X(N_X, N_Y)}{\partial N_X}\right|_{B_X^*} < 0, \tag{A23}$$

$$W_Y(r, 0) < 0, \tag{A24}$$

which gives:

$$\begin{aligned} r &> 0, \\ b + c &< 1, \end{aligned} \tag{A25}$$

where the former is obvious requirement. Therefore, we conclude that $b + c < 1$ leads to the local stability of species exclusion of B_X^*. Eqn (A25) is independent of species label and therefore applies to both species. Hence the boundary equilibrium B_X^* is stable if and only if B_Y^* is stable (which is obvious because we have assumed that X and Y are ecologically equivalent). Coexistence state $C^* = (N_X^*, N_Y^*)$ occurs when the leading real part of the eigenvalue of the Jacobi matrix ($\mathcal{J}$) around C^* is negative. It is impossible to derive the analytical expression for the stability condition, and hence we carried out numerical investigation for the stability.

D Numerical procedures for basins of attraction

Using Mathematica (Wolfram Research 2018), we visualized the dynamics in the main text. We outline its minimal procedure. Readers may want to use Module, Block, or With, if tuning parameters is preferred.

First, define a map (or 'flow') $\big(t, N_X(0), N_Y(0)\big) \mapsto \big(N_X(t), N_Y(t)\big)$; second, numerically obtain $W_X(N_X^*, N_Y^*) = W_Y(N_X^*, N_Y^*) = 0$ for given parameter values; it is of use to issue $W_X\big(N_X^*, 0\big) = 0$ for B_X^* and $W_Y\big(0, N_X^*\big) = 0$ for B_Y^*. Then assess which equilibrium a given initial condition (say, $(r/2, r/2)$) converges to; depending on the convergence fates, we separate the region using `RegionPlot` function. Stability is determined by `EigenValues` function; depending on the sign of the real part of the eigenvalues, we can depict stable or unstable equilibrium as a disk or closed circle, respectively, using `If` function.

E General case

Effects of $p_{X|Y}$ and $p_{Y|X}$

From behavioral observation data, we used highly asymmetric parameter sets:

$$p_{X|X} = q_{X|X} = 0.4, \; p_{X|Y} = 0.8; \tag{A26}$$

$$p_{Y|Y} = q_{Y|Y} = 0.8, \; p_{Y|X} = 0.4. \tag{A27}$$

However, when these values are relatively symmetric, the community dynamics consequences are more divergent. In particular, with symmetry in p's and q's, there would be a bi-stability (Kuno 1992; Kishi & Nakazawa 2013; Kyogoku & Sota 2017).

For the completeness, we here illustrate the effects of symmetry in p's and q's on the community dynamics by plotting phase portraits. To this end, it is useful to reduce the parameters by the following equations:

$$\begin{aligned} E_X &= (1-c)r + cr\frac{f_X}{f_X + \frac{p_{X|Y}}{p_{X|X}} f_Y}\left(1 + \frac{p_{X|Y}}{p_{X|X}} q_{X|X} f_Y\right), \\ E_Y &= (1-c)r + cr\frac{f_Y}{f_Y + \frac{p_{Y|X}}{p_{Y|Y}} f_X}\left(1 + \frac{p_{Y|X}}{p_{Y|Y}} q_{Y|Y} f_X\right) \end{aligned} \tag{A28}$$

so that we can subsume the fractions into compound parameters:

$$\frac{p_{X|Y}}{p_{X|X}} = \pi_{X|Y}, \; \frac{p_{Y|X}}{p_{Y|Y}} = \pi_{Y|X} \tag{A29}$$

that represent relative acceptance rate of a female for heterospecific males over conspecific males. To look at the effect of acceptance rates of females ($\pi_{X|Y}$ and $\pi_{Y|X}$), we fix $q := q_{X|X} = q_{Y|Y}$ at a particular value from 0, 0.5, to 1, and vary π's (Fig. A 2).

As we have found already, the stability for boundary equilibria (B_X^*, B_Y^*) is independent of π's and q, while that for interior equilibria depends on π's and q. As such, varying π's and q can generate the bistability of species exclusion and coexistence.

Differential mating activity

The rate of mating attempt in a given time (hereafter mating activity) can differ remarkably between species (see Fig. 3 in the main text). To account for this, we define asymmetric frequencies g_X and g_Y by:

$$g_X = \frac{N_X}{N_X + \alpha_Y N_Y} = 1 - g_Y, \tag{A30}$$

where α_{Y} represents the relative mating activity of a Y-male over X-male ($\alpha_{\mathrm{Y}} > 0$). Higher α_{Y} indicates a higher mating activity of Y-males; $\alpha_{\mathrm{Y}} = 1$ implies the even level of mating activity.

We replaced f's by g's:

$$\begin{aligned}
E_{\mathrm{X}} &= (1-c)r + cr\frac{g_{\mathrm{X}}}{g_{\mathrm{X}} + \frac{p_{\mathrm{X|Y}}}{p_{\mathrm{X|X}}} g_{\mathrm{Y}}} \left(1 + \frac{p_{\mathrm{X|Y}}}{p_{\mathrm{X|X}}} q_{\mathrm{X|X}} g_{\mathrm{Y}}\right), \\
E_{\mathrm{Y}} &= (1-c)r + cr\frac{g_{\mathrm{Y}}}{g_{\mathrm{Y}} + \frac{p_{\mathrm{Y|X}}}{p_{\mathrm{Y|Y}}} g_{\mathrm{X}}} \left(1 + \frac{p_{\mathrm{Y|X}}}{p_{\mathrm{Y|Y}}} q_{\mathrm{Y|Y}} g_{\mathrm{X}}\right),
\end{aligned} \tag{A31}$$

and found that increasing α_{Y} leads to higher possibility of the extinction of X; this is a natural result because mating activity can lead to higher heteropecific mating-chance for X-females. Obviously there are other ways to incorporate differential activities depending on behavioral aspects.

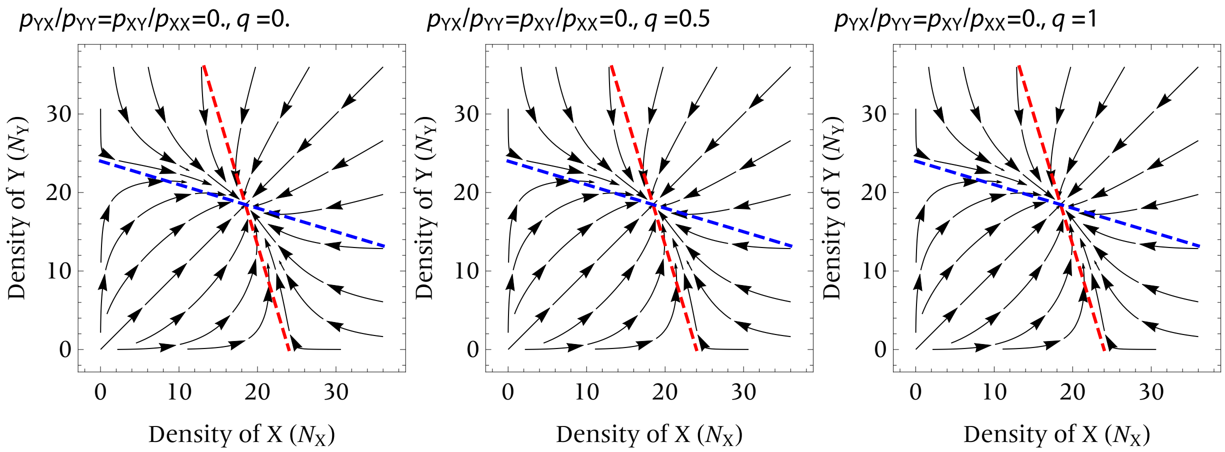

Fig. A1: Phase portraits, with $\pi_{Y|X} = \pi_{X|Y} = 0$ and $q = 0, 0.5, 1$ (from left to right). Because $\pi_{Y|X} = \pi_{X|Y} = 0$ implies that heterospecific mating is impossible, RI does not occur and thus the two species coexist. Note that q does not matter, because heterospecific mating is excluded.

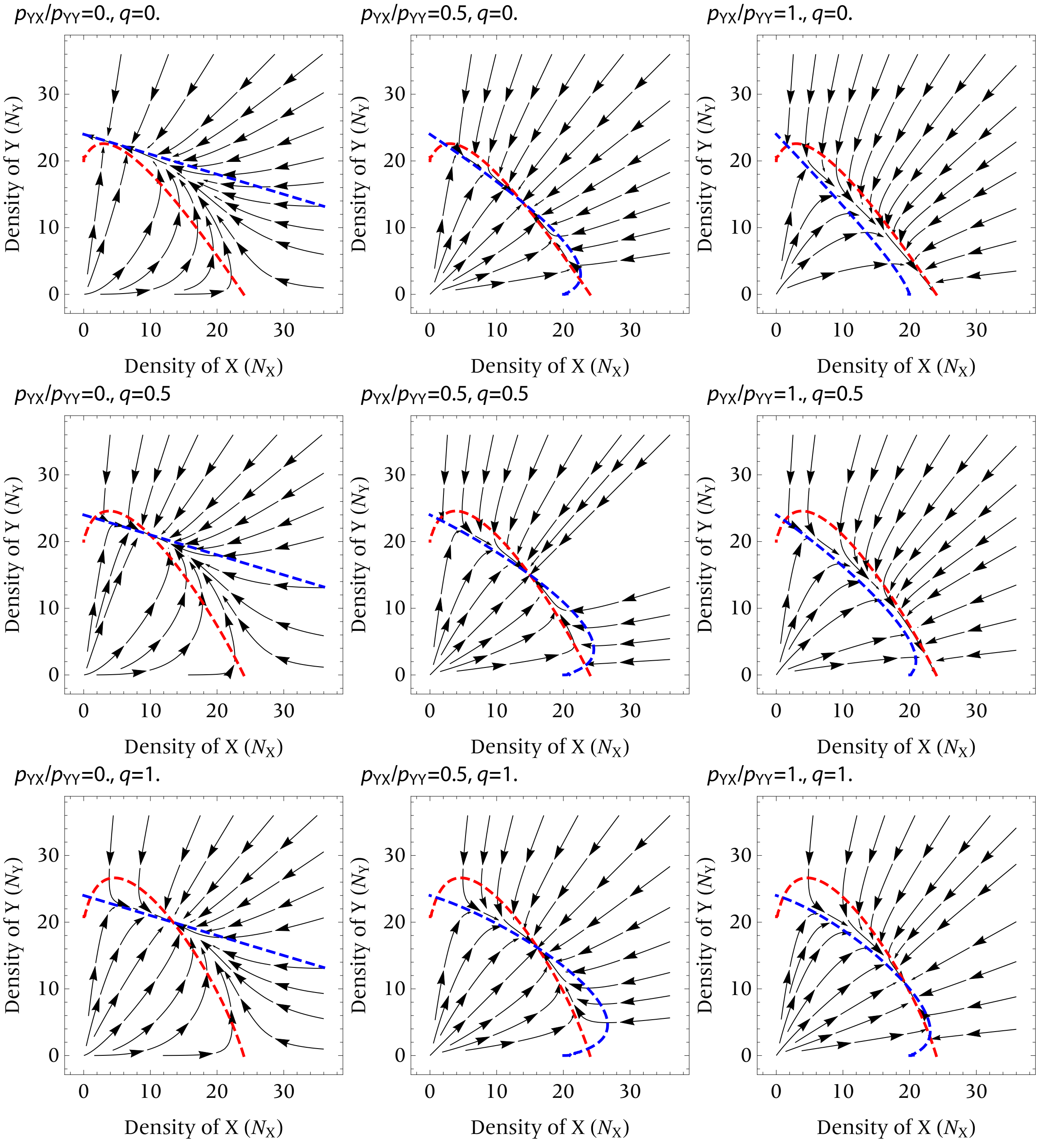

Fig. A2: Phase portraits, with $\pi_{Y|X} = 0, 0.5, 1$ (from left to right) and $q = 0, 0.5, 1$ (from top to bottom), given $\pi_{X|Y} = 0.5$ (fixed). From these panels, we can see that bistabitliy can occur. Red curves: isoclines for X; Blue curves: isoclines for Y.

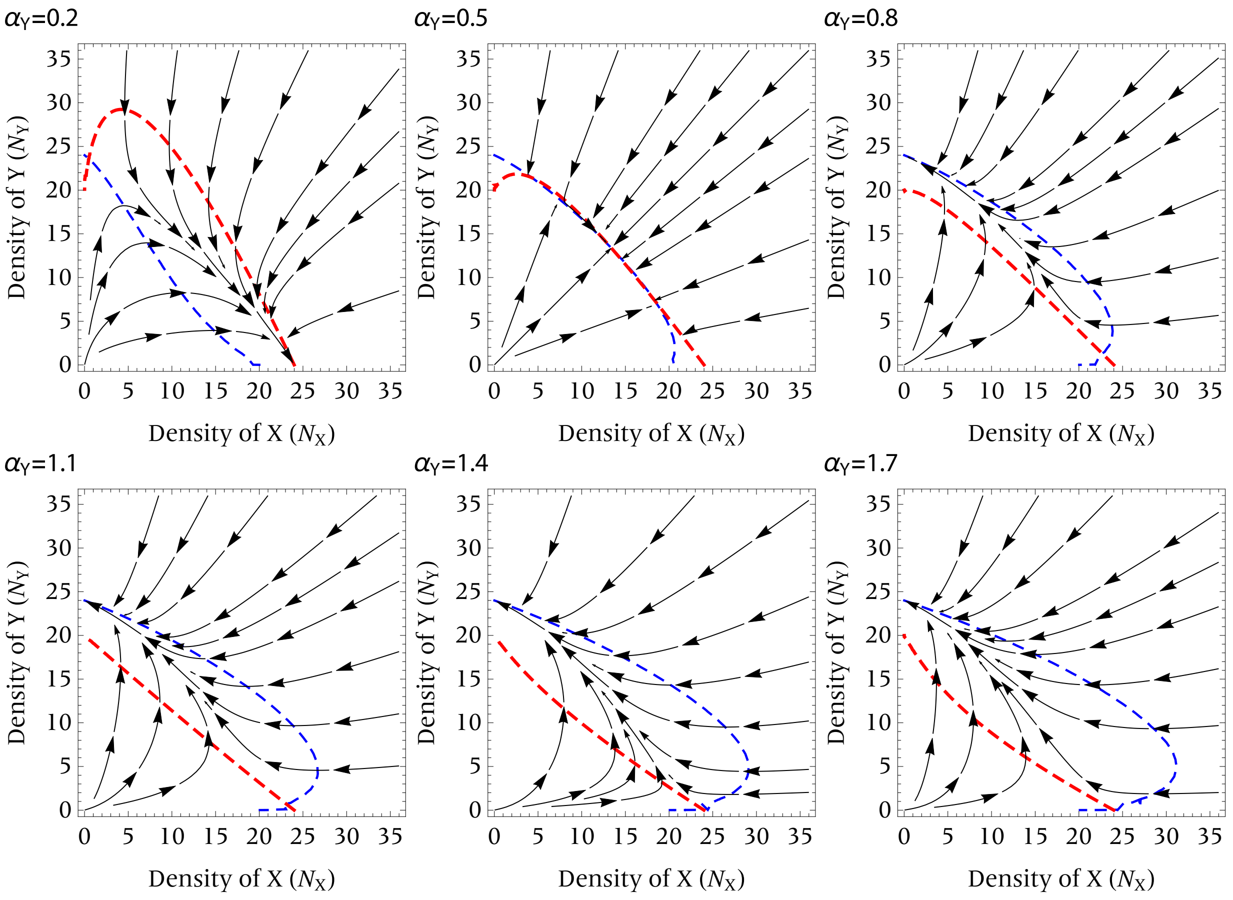

Fig. A3: Isoclines and phase portraits for the community dynamics. Red curves: isoclines for X; Blue curves: isoclines for Y. Higher α_Y leads to lower possibility of the persistence of X. Default parameter values are used.

References

Kishi, S. & Nakazawa, T. (2013). Analysis of species coexistence co-mediated by resource competition and reproductive interference. *Population Ecology*, **55**.2, pp. 305–313. DOI: 10.1007/s10144-013-0369-2.

Kuno, E. (1992). Competitive exclusion through reproductive interference. *Researches on Population Ecology*, **34**.2, pp. 275–284. DOI: 10.1007/BF02514797.

Kyogoku, D. & Sota, T. (2017). A generalized population dynamics model for reproductive interference with absolute density dependence. *Scientific Reports*, **7**. DOI: 10.1038/s41598-017-02238-6.

Majerus, M. E. (2003). *Sex wars: genes, bacteria, and biased sex ratios*. Princeton University Press.

Niijima, K, Tobita, M, & Matsuka, M (2000). Development of low-cost and laborsaving technique for group rearing of a ladybird, Harmonia axyridis. *Tamagawa University Research Review*, **6**, pp. 23–33.

Noriyuki, S. (2016). Functional convergence and phenotypic divergence in two specialist species of pine-associated ladybirds. *Evolutionary Ecology*, **31**.6, pp. 885–898. DOI: 10.1007/s10682-017-9918-z.

Noriyuki, S., Kameda, Y., & Osawa, N. (2014). Prevalence of male-killer in a sympatric population of two sibling ladybird species, Harmonia yedoensis and Harmonia axyridis (Coleoptera: Coccinellidae). *European Journal of Entomology*, **111**.2, pp. 307–311. DOI: 10.14411/eje.2014.029.

Okada, I, Niijima, K, & Toriumi, Y (1978). Comparative studies on sibling species of lady beetles, *Harmonia yedoensis* and *H. axyridis* (Coleoptera: Coccinellidae)[insects]. *Bulletin of the Faculty of Agriculture Tamagawa University*,

Wolfram Research, I. (2018). *Mathematica, Version 11.3*. Champaign, IL.
